# Complex cytogenetic abnormalities in chronic myeloid leukemia resulting in early progression to blast crisis: a case report

**DOI:** 10.1186/s13256-020-02539-x

**Published:** 2020-11-27

**Authors:** Haider Ali Malakzai, Soma Rahmani, Ahmed Maseh Haidary, Sarah Noor, Maryam Ahmad, Abdul Sami Ibrahimkhil, Samuel Sharif

**Affiliations:** 1 Department of Pathology and Laboratory Medicine, French Medical Institute for Mothers and Children (FMIC), Kabul, Afghanistan; 2 Department of Haemato-Oncology, Jumhoriat Hospital, Ministry of Public Health, Kabul, Afghanistan

**Keywords:** CML, Complex cytogenetic abnormalities, Early progression, Nonresponsive to therapy

## Abstract

**Introduction:**

BCR-ABL1, resulting from t(9;22), is the oncogenic driver of chronic myeloid leukemia and the therapeutic target of the disease. Molecular studies have been the gold standard modality for patient assessment since the advent of tyrosine kinase inhibitor therapy. In spite of that, there are cytogenetic abnormalities that can render the disease unresponsive to conventional therapy, thus making cytogenetics an important component of patient management guidelines.

**Case presentation:**

We present a case of a Tajik, Afghan patient with chronic myeloid leukemia with del(6)(q23.3q27), t(9;22)(q34;q11.2), monosomy 11, monosomy 12, and marker chromosome who, despite having typical clinical and hematological disease with initial response to therapy, progressed to blast crisis very early and thus required special interventions.

**Conclusion:**

Cytogenetic monitoring is an important pillar in the management of patients with chronic myeloid leukemia that cannot be ignored. It should therefore be a part of patient management not only during diagnosis but also during management. We present an unusual cytogenetic abnormality in a patient with chronic myeloid leukemia that resulted in early disease progression.

## Introduction

BCR-ABL1, the oncogenic driver of chronic myeloid leukemia (CML), results from a balanced translocation, t(9;22)(q34;q11.2), involving a fusion of the Abelson gene (*ABL*) from chromosome 9q34 with the breakpoint cluster region (*BCR*) gene on chromosome 22q11.2 [1]. This fusion gene is the main molecular target in the management of CML, and, since its first introduction in early 2000s, tyrosine kinase inhibitor (TKI) therapy has been very effective in improving outcome and prognosis in affected patients [2]. The first agent with TKI activity that acquired license for treatment of patients with CML was imatinib [3]. Then the new-generation drugs were added, including nilotinib, dasatinib, and ponatinib [4].

The current therapeutic protocols are all based on cytogenetic and molecular genetic predictors of disease [1, 5]. For instance, the first tool to identify patient response with a great level of confidence was quantitative polymerase chain reaction (PCR) for BCR-ABL1, aiming to assess patients for major molecular response, which was the deepest scrutiny into a disease process for its time [6]. Since then, scrutiny of the disease process has grown deeper and deeper, with more agile and accurate methods of identifying abnormal clones with ratio of 1:10,000 versus normal clones, called the deep molecular response (DMR) [7]. Now, patients who have achieved DMR and are able to maintain it for a specific time period are even entitled to be allowed complete discontinuation of TKI therapy [8].

In spite of all the above-mentioned molecular progress in diagnosis and monitoring of CML, the role of cytogenetics is still undeniably very significant because molecular modalities, though very useful, might still be unable to identify extra Philadelphia chromosome (Ph) abnormalities, including the major route cytogenetic abnormalities that can affect management as well as prognosis [9]. We present a case of a patient with CML who was diagnosed in chronic phase (CP) and subsequently started on TKI therapy with initial hematological response; however, within 5 months, the patient experienced a blast crisis due to acquisition of extra Ph abnormalities.

## Case presentation

**Fig. 1:**
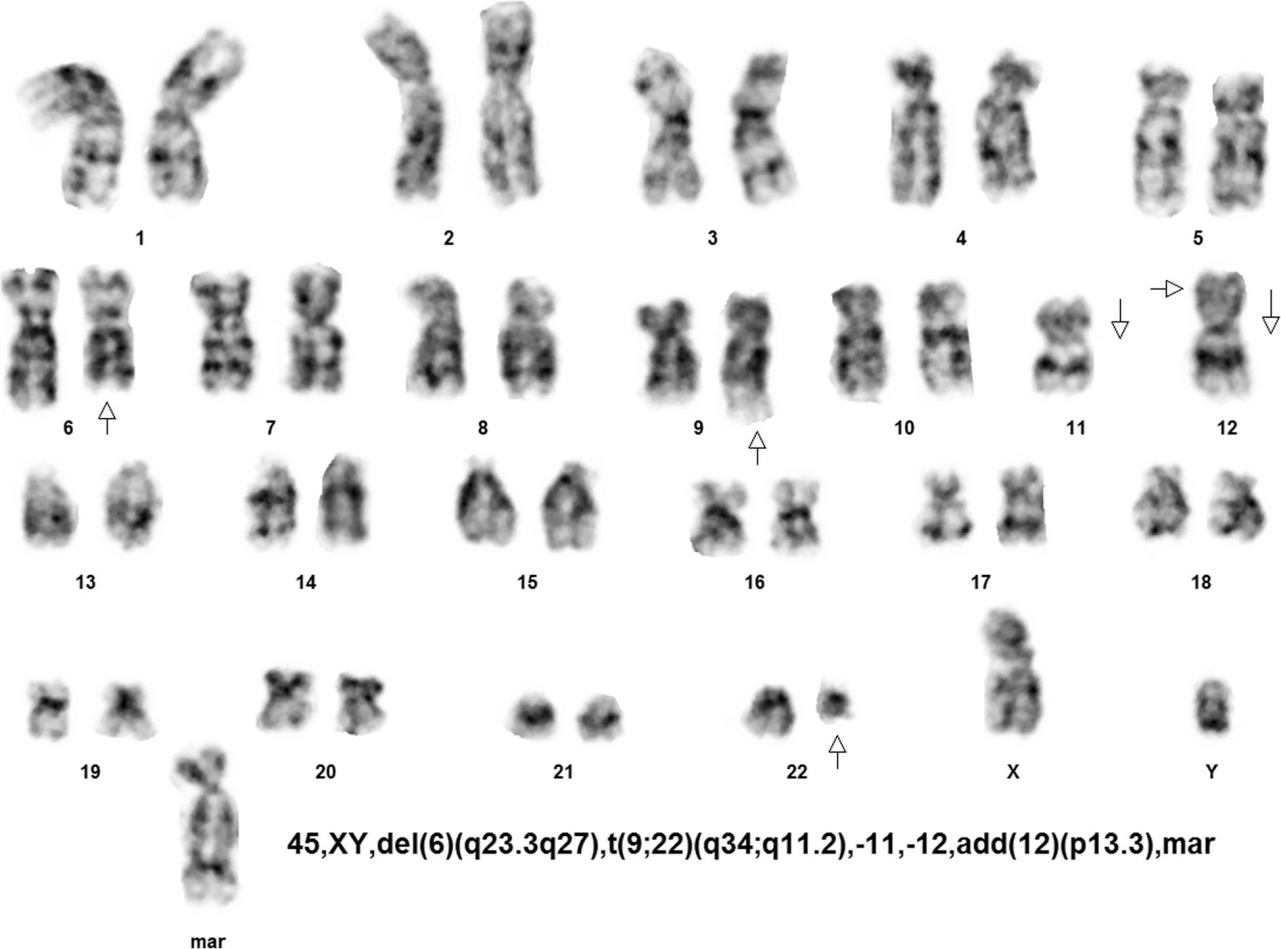
Karyogram of one of the two cells assessed, showing 45,XY with del(6)(q23.3q27), t(9;22)(q34;q11.2), monosomy 11, monosomy 12, and marker chromosome labeled as (mar)

Our patient was a 42-year-old Tajik, Afghan man with no known medical illness who presented to our institution with lethargy, anorexia, pallor, and progressive abdominal distension that had developed over a duration of 4 months. On examination, the patient was pale, not in distress, and had no lymphadenopathy. His abdominal examination revealed gross splenomegaly crossing the midline with the liver just two finger breadths in the subcostal region. His complete blood count revealed moderate anemia, thrombocytosis, and hyperleukocytosis (450,000 white blood cells per microliter), showing predominance of granulocytes with a bimodal peak of mature neutrophils (68%) and myelocytes (33%). The patient’s blast count was 4% with normal basophil count. The clinical impression at that time was CML in CP based on the initial clinical and hematological evaluation. The patient was counseled to proceed with cytogenetic analysis for confirmation and prognostication of disease, but, due to financial constraints, he opted to start on TKI therapy. Accordingly, the patient was started on conventional imatinib therapy 400 mg/day. Initially, he responded well with resolution of hyperleukocytosis and improvement in hemoglobin and platelet count toward normal, but he did not achieve complete hematological remission. A repeated complete blood count analysis revealed moderate anemia, mild thrombocytopenia, and the presence of >90% blast cells in a total white cell count of 62,000/μl. After detailed counseling, the patient agreed to proceed with cytogenetic analysis, in which the analyzed metaphases of all 20 cells revealed 45,XY, presence of Ph chromosome (9q34;22q11.2), and additional complex chromosomal abnormalities, including deletion of chromosomes 6q23.3 to q27, monosomy 11, monosomy 12, insertion 12p13.3, and a marker chromosome, as shown in Fig. 1. Ultimately, the patient and his family were advised to proceed with allogeneic bone marrow transplant, considering the patient’s complex karyotype and nonresponsiveness to TKI therapy.

## Discussion

CML, once an indolent but indefinitely progressive disease of the hematopoietic system, has now become a success story in the field of hemato-oncology [10]. Almost all patients now achieve complete remission in a matter of months, and this success is attributable to the discovery of TKI agents and progress in the field of molecular genetics [11, 12].

The field of molecular genetics has enabled physicians not only to identify the disease burden but also to tailor management in accordance with measurable residual disease [1, 13]. All the guidelines since the advent of quantitative real-time PCR for BCR-ABL1, just two decades ago, include close patient monitoring with consideration of molecular targets [9, 14]. Quantitation of disease burden with consideration of established guidelines not only has enabled identification of patients for whom TKI therapy needs to be switched from one agent to a more potent one during therapy but also has made it possible to identify patients for whom a higher class of TKI must be considered right from the start [15].

Targeting deep molecular response has enabled identification of individuals who can successfully achieve complete molecular remission with successful discontinuation of therapy [8]. Novel, highly sensitive quantitative PCR analysis methods are now implemented to monitor patients who discontinue TKI therapy after successful achievement of molecular targets [16].

Mutational studies have now become a commonplace practice in the management of patients with CML [10]. The presence of mutation(s) of bad prognostic value implies that the patient needs commencement with expensive but more specific tyrosine kinase domain targeting agents, such as dasatinib and ponatinib [12, 17]. In such cases, it has been demonstrated that ponatinib, the newest agent in the market, is the agent that is effective in almost all the mutant forms of BCR-ABL1 demonstrated so far [18].

Despite all the mentioned progression in molecular studies, cytogenetics plays a very important role and thus has always been incorporated in CML management guidelines [19]. Chromosomal abnormalities that are acquired during either the pathogenesis or the treatment of CML have a deep impact on disease progression, prognosis, and thus shift the paradigm of management. Although even patients with the mutations of worst prognostic significance can benefit from third-generation TKI therapy, such as ponatinib, patients who harbor cytogenetic markers of prognostic significance have to be considered for alternative therapeutic modalities, especially allogeneic bone marrow stem cell transplant [20]. This is because it has been demonstrated that such clones that acquire additional cytogenetic abnormalities are resistant to conventional TKI inhibitor therapy [19].

Our patient, despite having the characteristic clinical and hematological profile of CML, progressed to blast crisis early during therapy for harboring complex chromosomal abnormalities that were in addition to the pathognomonic Ph. Thus, the role played by cytogenetic analysis in the management of patients with CML along with the modern molecular modalities is absolutely not replaceable. Therefore, when feasible, cytogenetic studies must be included in the analysis of patients for whom molecular tests are being done.

## Conclusion

We present a case of a patient with CML who had an unusual “complex” cytogenetic abnormality in addition to Ph that resulted in early blast crisis. To our knowledge, this is the first case report of a patient with CML harboring del(6)(q23.3q27), t(9;22)(q34;q11.2), monosomy 11, monosomy 12, and marker chromosome in addition to Ph.

## Data Availability

All the generated data are included in this article.

## Abbreviations

ABL1: Abelson gene 1
BCR: Break point cluster region
CML: Chronic myeloid leukemia
CP: Chronic phase
PCR: Polymerase chain reaction
Ph: Philadelphia chromosome
TKI: Tyrosine kinase inhibitor
